# Trace-element XAFS sensitivity: a stress test for a new XRF multi-detector

**DOI:** 10.1107/S1600577521008857

**Published:** 2021-10-18

**Authors:** Ilaria Carlomagno, Matias Antonelli, Giuliana Aquilanti, Pierluigi Bellutti, Giuseppe Bertuccio, Giacomo Borghi, Giuseppe Cautero, Daniela Cirrincione, Giovanni de Giudici, Francesco Ficorella, Massimo Gandola, Dario Giuressi, Daniela Medas, Filippo Mele, Ralf H. Menk, Luca Olivi, Giulio Orzan, Antonino Picciotto, Francesca Podda, Alexandre Rachevski, Irina Rashevskaya, Luigi Stebel, Andrea Vacchi, Gianluigi Zampa, Nicola Zampa, Nicola Zorzi, Carlo Meneghini

**Affiliations:** a Elettra Sincrotrone Trieste, Basovizza, Trieste, Italy; b INFN – Trieste, Padriciano, Trieste, Italy; c Fondazione Bruno Kessler – FBK, Trento, Italy; d TIFPA – INFN, Trento, Italy; e Politecnico di Milano, Como, Italy; f INFN – Milano, Milano, Italy; g Department of Mathematics, Computer Science, and Physics, University of Udine, Udine, Italy; h Department of Chemical and Geological Science, University of Cagliari, Cagliari, Italy; i Department of Medical Imaging, University of Saskatchewan, Saskatoon, Canada SK S7N 5A2; j Department of Science, University of Roma Tre, Roma, Italy

**Keywords:** X-ray fluorescence, fluorescence detector, X-ray absorption spectroscopy, geology

## Abstract

X-ray absorption fine-structure (XAFS) spectroscopy can assess the chemical speciation of the elements providing their coordination and oxidation state, information generally hidden to other techniques. In the case of trace elements, achieving a good quality XAFS signal poses several challenges, as it requires high photon flux, counting statistics and detector linearity. Here, a new multi-element X-ray fluorescence detector is presented, specifically designed to probe the chemical speciation of trace 3*d* elements down to the p.p.m. range. The potentialities of the detector are presented through a case study: the speciation of ultra-diluted elements (Fe, Mn and Cr) in geological rocks from a calcareous formation related to the dispersal processes from Ontong (Java) volcanism (mid-Cretaceous). Trace-elements speciation is crucial in evaluating the impact of geogenic and anthropogenic harmful metals on the environment, and to evaluate the risks to human health and ecosystems. These results show that the new detector is suitable for collecting spectra of 3*d* elements in trace amounts in a calcareous matrix. The data quality is high enough that quantitative data analysis could be performed to determine their chemical speciation.

## Abstract 2

A new multielement X-ray fluorescence detector is presented, specifically designed to probe the chemical speciation of trace 3*d* elements down to the p.p.m. range.

## 1. Introduction

X-ray absorption spectroscopy (XAS) is an element-selective technique, sensitive to the local coordination chemistry and to the valence state of the absorber, independently from its physical state, crystallographic phase (crystalline, amorphous) or morphology (Koningsberger & Prins, 1988 ▸; Parsons *et al.*, 2002 ▸; Mastelaro & Zanotto, 2018 ▸; Kuzmin & Chaboy, 2014 ▸). XAS can be applied to studies over a wide range of temperature (from a few K to some thousands of K) and pressure (Koningsberger & Prins, 1988 ▸; Bunker, 2010 ▸). Owing to these characteristics, XAS is widely exploited in geological and environmental sciences where the materials of interest are often highly heterogeneous both in chemical composition and physical status, presenting mixtures ranging from crystalline to amorphous phases (see Quartieri, 2015 ▸; De Giudici *et al.*, 2015 ▸; Brown Jr & Sturchio, 2002 ▸; Jones, 1999 ▸, and references therein). The possibility to identify the chemical species to which the elements belong is one of the major strengths of the XAS technique when dealing with environmental science: in fact, the impact of an element in terms of hazardousness, for example, is determined not only by its concentration but also, crucially, by its chemical nature (Tack & Verloo, 1995 ▸). The latter also determines the element bioavailability, mobility, pathways and toxicity (Tack & Verloo, 1995 ▸; Hettiarachchi *et al.*, 2017 ▸), thus affecting specific ecosystem functions and components. Therefore, chemical speciation is key information to understand environmental risks to human health and ecosystems, and can help in the design of remedial actions to prevent the dispersion of pollutants (Scheckel & Ford, 2010 ▸). In paleoenvironmental science, the variability of trace elements in rocks is a useful proxy for insights into geological events such as regional dispersion of 3*d* metals over paleoceans and disruptive volcanic events (Kravtsova, 2020 ▸; Terzano *et al.*, 2019 ▸). For this reason, the simultaneous assessment of chemical concentration and mineralogical speciation of trace elements, possibly carried out in unique analytical routines, is crucial to understand environmental processes.

Synchrotron radiation X-ray fluorescence (SR-XRF) is widely used to individuate and quantify the elements in samples. Compared with SR-XRF, XAS in fluorescence mode poses additional challenges due to the nature of the signal itself, represented by the fine-structure oscillations (XAFS) of the absorption coefficient appearing in the near-edge (XANES) and extended post-edge region (EXAFS) (Bunker, 2010 ▸). The XANES features generally represent roughly 10% of the total XAS signal of the absorbing element while, at higher energies, the EXAFS features may decrease even below the 1% of the absorption signal. Therefore, measuring the XAFS signal requires much stricter specifications to the detection system than those needed for XRF. In particular, when the element of interest is highly diluted, as in the case of trace elements, the Poisson noise associated with fluorescence photons emitted from the atoms of the matrices represents a major obstacle to the XAFS measurement.

A possible approach to cope with these constraints is to increase the incident X-ray photon flux (high counts) and to use monochromators on the fluorescence yield (crystal analyzers) to sharply select the absorption signal of the element of interest, as implemented on insertion device beamlines (Diaz-Moreno *et al.*, 2018 ▸). However, the high brilliance of the X-ray beams may induce radiation damage in photosensitive systems (*e.g.* oxides of Fe and Mn) and organic materials, while the high energy resolution provided by the analyzers (Δ*E*/*E* < 10^−3^) is often not required for the purpose of XAS experiments, making them more suitable for specific techniques such as resonant inelastic X-ray scattering (RIXS) (Brookes *et al.*, 2018 ▸). Solid state detectors based on Si and Ge semiconductors possess a more suitable energy resolution (Δ*E*/*E* ≃ 10^−2^) (Cramer *et al.*, 1988 ▸; Fabjan & Schopper, 2020 ▸; Chatterji *et al.*, 2016 ▸) and represent a valuable alternative. In particular, Si detectors using the sideward depletion method such as silicon drift detectors (SDDs) (Gatti & Rehak, 1984 ▸; Gatti *et al.*, 1984 ▸) enable spectroscopic measurements with an energy resolution close to the Fano limit (Fano, 1947 ▸) when coupled to custom ultra-low-noise front-end electronics (Mele *et al.*, 2021 ▸). However, especially for diluted samples, the yield of specific fluorescence lines of the trace elements might be low, while the overall photon flux impinging on the detector, mainly originating from the elastic and inelastic scattering and the fluorescence yield from the matrix, is usually extremely high. In such situations, the use of multi-element detectors is indispensable and of paramount importance since single, albeit large, area spectroscopic detectors would suffer from pile-up and saturation effects. In addition, microcrystalline phases in specimens can give rise to Bragg peaks that spoil the spectra with spikes, glitches or even paralyze the detector at given energies. Dividing the acquisition area into independent elements is a suitable way to cope with these problems. If Bragg peaks affect few detector elements at selected energies, the signal of the saturated elements can be removed during post-processing analysis (Meneghini *et al.*, 2009 ▸). However, when intense Bragg peaks excite the total fluorescence of the sample so affecting the signal of several detector elements, then the multidetector geometry does not help with respect to other solutions. A number of custom-made and commercial off-the-shelf detector systems are already available and, for most of them, the manufacturing companies (such as, but not limited to, Mirion, Hitachi and Ketek) offer multi-element solutions (4–32 elements) based on individual SDDs, while PNsensor provides also monolithic multi-channel silicon sensors (Strüder, 2000 ▸). Regarding Ge detectors, Canberra (now Mirion Technology) has provided for instance 32 and 64 channel detectors (Chatterji *et al.*, 2016 ▸), and is currently offering high-purity monolithic Ge detectors with up to 64 elements. Although the application of off-the-shelf detectors in XAS experiments in fluorescence mode is a convenient approach, only custom tailored detector systems are able to thoroughly exploit the potential of state-of-the-art beamlines (Thompson *et al.*, 2000 ▸). The MAIA detector for instance (Ryan *et al.*, 2014 ▸; Chen *et al.*, 2017 ▸) is an excellent example of a custom-made solution featuring an array of 20 × 20 individual silicon diodes or small-sized silicon drift detectors with 1 mm^2^ active area. Another system developed by academic and industrial partners is the ARDESIA detector, based on a monolithic array of four circular or squarish SDDs (collimated active area of 12.56 mm^2^ or 15 mm^2^), which has been successfully installed and tested at different synchrotron radiation beamlines (Bellotti *et al.*, 2016 ▸; Hafizh *et al.*, 2019 ▸).

In this framework, several Italian institutions have started collaborating on the design of a multi-element SDD detector tailored for the specific needs of the XAFS beamline at Elettra Sincrotrone Trieste (Di Cicco *et al.*, 2009 ▸). The research has focused on the study of ultra-diluted systems through XAS in fluorescence mode and aimed at achieving: (i) quantum efficiency higher than 80%, in particular in the low-energy region of the beamline (2.4–10 keV), (ii) both room-temperature operation and cooling of the sensors, suitable for automated beam time use, (iii) coverage of a wide solid angle of ≥30% at 10 cm from the sample, (iv) energy resolution Δ*E*/*E* ≤ 180 eV with peaking time of 1 µs. The sensors are produced by the Fondazione Bruno Kessler (FBK), a silicon foundry in Trento, Italy, that has developed a special production process yielding a substantial reduction of leakage currents in the sensors allowing the operation of large area detectors with excellent energy resolution even at room temperature (Bertuccio *et al.*, 2015 ▸, 2016 ▸).

Here we discuss the application of this detector in a XAFS study of 3*d* metal traces (Fe, Mn and Cr) in a calcareous formation. The very high dilution of the elements of interest, in particular for Cr reaching the p.p.m. concentration, represents a valuable stress test for the device.

## 2. Experimental section

### 2.1. The multi-element XRF detector

**Figure 1:**
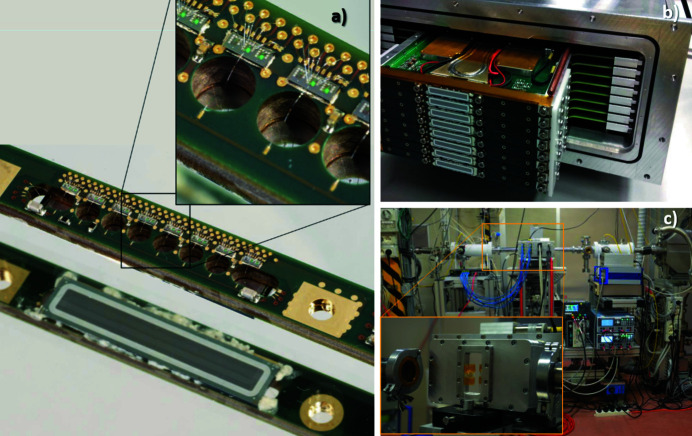
(*a*) One of the single modules of the detector described in the text. (*b*) Array of eight modules. (*c*) The detector mounted on the XAFS beamline.

At present, the multi-element detector (Rachevski *et al.*, 2019 ▸; Cirrincione, 2019 ▸; Bufon *et al.*, 2019 ▸) is assembled tiling up eight independent monolithic modules, each of which comprises eight linearly aligned SDDs (Gatti *et al.*, 1984 ▸) [Fig. 1 ▸(*a*)].

Each module is equipped with a Peltier cell and its own water pipeline to purge the residual heat of the former when the detector is operated in cooling mode. A new design technique (INFN, 2018 ▸) has been employed to maximize the active area of the sensors keeping the instrument as compact as possible. The sensors have been fabricated on 450 µm-thick, n-type high-purity silicon wafers with a resistivity of 9 kΩ cm. Each sensor module has typical dimensions of 26.65 mm × 5.65 mm, and it features eight SDD cells of 3 mm × 3 mm active area (0.478 area ratio). The active over total area ratio for the whole detector is 0.339. The size of the single SDD cell was decided on the basis of simulations that took into account not only the total active area but also the acquisition solid angle and the dead-time following each event. It has been noted that the increase in the number of cells, which divide the same total area, although initially dramatically increasing the efficiency, becomes less and less important when the dimensions of the cells are very small (also due to the dead zones introduced by the collimators and separations between cells). Therefore, a solution of 3 mm × 3 mm turned out to be the best compromise between efficiency optimization and constructive complexity of the system.

On the front side of each monolithic module a shallow and uniformly implanted p^+^ entrance window covers all eight SDD cells. This guarantees high efficiency even at very low photon energies down to and below the carbon *K*_α_ emission line at 277 eV (Bufon *et al.*, 2018 ▸, 2019 ▸). The back side of each SDD cell is segmented into concentric drift cathodes (p^+^ rings) and a small collecting anode (n^+^ pad) in the centre (Rachevski *et al.*, 2019 ▸; Cirrincione, 2019 ▸), which is virtually grounded through the attached front-end electronics. By using an integrated voltage divider, every drift cathode is biased with decreasing potential generating a gradient drift field, which is pointing away from the collecting anode. The interaction of X-ray photons with the bulk silicon creates electron–hole pairs. These charge carriers drift along the electric field lines, with the electrons being eventually collected at the anode. The number of generated electrons per photon, *i.e.* the released charge, is proportional to the characteristic energy of the X-ray photon. The photo-generated electrons are converted into a voltage proportional to the photon energy by the front-end electronics, connected to the readout anode via wire bonds. The front-end electronics is made up of custom-made, ultra-low noise, monolithic charge-sensitive pre-amplifiers operating in pulse reset mode (Bertuccio *et al.*, 2014 ▸). This system will be referred to as ‘SIRIO’ in the following. SIRIO enables low dead-time and high-count-rate measurements (some 10^6^ photons s^−1^) with energy resolution better than 150 eV FWHM at the Mn *K*_α_ emission line (5.9 keV) at room temperature. Each of the SIRIO output signals from a monolithic module is filtered and further amplified by CR-RC^2^ analog pre-shapers with selectable peaking time of 0.3 µs or 1.2 µs. The peak-shaped signals are then sampled by multichannel analog-to-digital converters (ADCs) with a resolution of 12 bits at 40 MHz sampling rate, and further filtered with trapezoidal finite impulse response (FIR) filters of variable length (peaking time ranges of 0.4–1.6 µs and 1.3–3.2 µs, respectively) in order to guarantee the maximum energy resolution even at a count rate of ∼2.5 × 10^5^ counts s^−1^ cell^−1^. Digital filtering, settings and complete monitoring of the instrument are realized within high-performance FPGAs, which send the acquired data to a host computer via a TCP/IP protocol. The detector discussed here employs a total of eight FPGA boards and eight TCP/IP transceivers. This modular structure allows an up- or down-scaling towards larger or smaller detection systems. In both cases, each individual cell is operated independently and includes different preset digital filters. The detector acquisition software ensures the proper alignment and calibration of the energy spectra for all the cells. Moreover, the software offers the possibility to separately save the information related to each cell (raw and pre-processed energy spectra) while its multichannel analyzers (MCAs) allow to select specific energy regions from the total XRF spectrum of each cell.

The system provides an efficient pile-up rejection and can identify the count rate excess on each cell accounting for the events that can be correctly analysed and ignoring the ones that should be rejected. The software also performs a pre­liminary data treatment on-the-fly, during the data acquisition, which includes the count rate dead-time correction, resulting in a clean, ready-to-use energy spectrum for each cell (Ciatto *et al.*, 2004 ▸). The detector read-out electronics coupled with the specifically developed software provides an automatic (rate-induced) or manually selected cell disabling feature allowing to prevent system paralysis in case of extremely high count rates (*e.g.* Bragg peaks).

**Figure 2:**
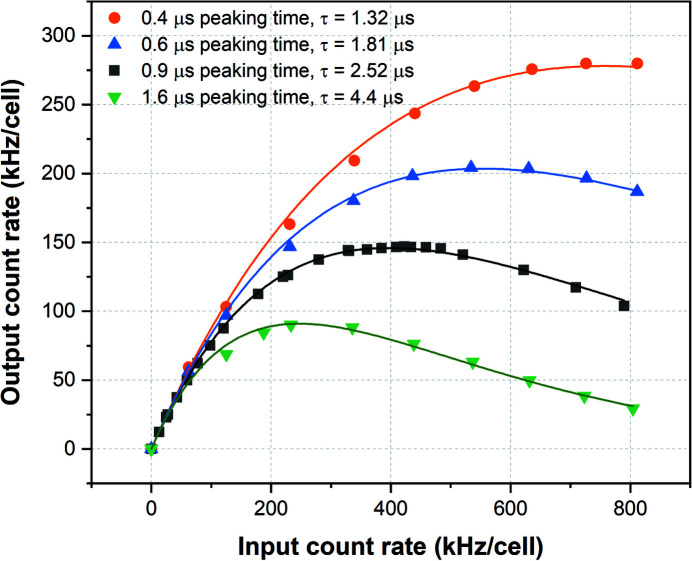
Output count rate versus input count rate for different peaking times adapted from Bufon *et al.* (2019 ▸) for a single cell. Considering the entire detector has 64 cells, the system can handle more than 10 Mcounts s^−1^ within a linearity of 75%. The symbol τ indicated in the annotation is the fitted dead-time in the model of a paralyzed detector.

**Figure 3:**
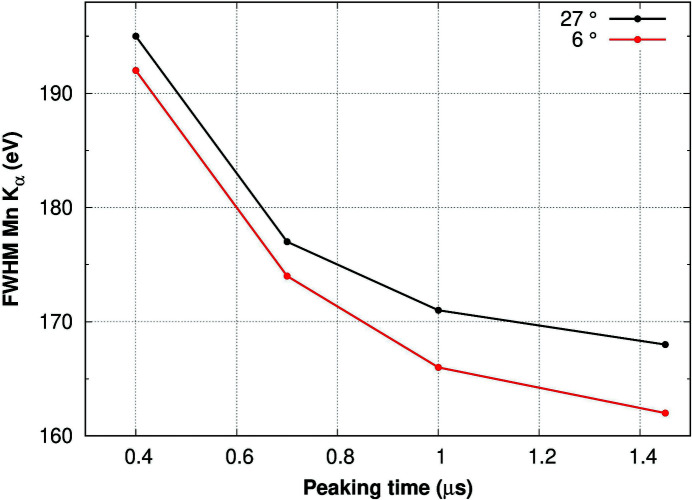
Energy resolution versus peaking time at room temperature (black line) and for the cooled detector (red line) with an average temperature of the sensors of 27°C and of 6°C, respectively.

As depicted in Fig. 2 ▸, the high rate behaviour follows that of a paralyzed detector, which is higher than that reported in data sheets of the majority of commercial devices or reported for custom-made solutions considering the maximum output count rate ( Ryan *et al.*, 2014 ▸) but somewhat lower than what is reported by Hafizh *et al.* (2019 ▸), which operates at shorter peaking times (≤128 ns). In Fig. 3 ▸ the energy resolution (FWHM) for the Mn *K*_α_ emission line at 5.9 keV is shown as a function of the peaking time, revealing little difference between the cooled detector (red line) and room-temperature operation (black line), demonstrating the very low noise level associated with the SDD leakage current. While the room-temperature behaviour is unique in comparison with other multi-element detectors, the energy resolution for the cooled detector is still somewhat inferior to that reported by Strüder (2000 ▸) and Bellotti *et al.* (2016 ▸). Furthermore, the fact that the distal cells are more prone to Compton contributions compared with the centre cell can be exploited for morphological studies of the sample (Billè *et al.*, 2016 ▸).

### 2.2. Samples and chemical analysis

The samples for our case study were collected on a calcareous formation in Cava Colonella (so-called chalkstone formation di Bari) in Murge, Apulia, Italy (Istituto Superiore per la Protezione e la Ricerca Ambientale, 2008 ▸). This geological formation is related to the Ontong Java volcanism which is associated with the global environmental perturbations in the mid-Cretaceous. In samples like these, the possibility to understand the speciation of some 3*d* metals like Fe, Mn and Cr is specifically useful to mark dispersal processes from these geological events.

For chemical analysis, samples were ground in an agate mortar. Acid digestion was carried out on 0.2 g of each sample by the microwave ETHOS One, Advanced Microwave Digestion System, Milestone. A high-purity mixture of 4 ml of Milli-Q water (<0.1 µS cm^−1^) and 6 ml of HCl (37% Suprapur) was added to the solids into microwave vessels. Samples were processed together with a blank and a reference material prepared with the same mixture. After cooling, the mixtures were transferred into Teflon beakers rinsing the vessels with a few ml of Milli-Q water. These mixtures were heated in a hot-plate (110°C); following evaporation, 3 ml of concentrated HNO_3_ (65% Suprapur) were added three times. Finally, the mixture was filtered (0.4 µm), and the solution was made up to 50 ml final volume using Milli-Q water. Trace elements were quantified by inductively coupled plasma optical emission spectrometry (ICP-OES; ARL Fisons ICP Analyzer 3520 B), and inductively coupled plasma mass spectrometry (ICP-MS; PerkinElmer, Elan/DRC-e, USA). To evaluate the accuracy (<5%) and precision (<5%) of the chemical analysis, EnviroMAT Drinking Water (SCP Science), EP-H-3 and EP-L-3 reference solutions were used. Concentrations of Fe, Mn and Cr are reported in Table 1 ▸.

### 2.3. XAFS measurements and data treatment

The XAS measurements for the presented case study were collected at the XAFS beamline (Di Cicco *et al.*, 2009 ▸) at Elettra synchrotron radiation facility. The multi-element detector was installed in the experimental hutch [Fig. 1 ▸(*c*)] oriented at 90° with respect to the incoming beam in the horizontal plane, in order to minimize the elastic scattering signal from the sample. An Al filter was placed in front of the detector in order to attenuate the intense background fluorescence signal coming from the lighter element (mainly Ca) matrix. The Al filter allowed the detector count rate to remain above the 90% linearity response region.

**Figure 4:**
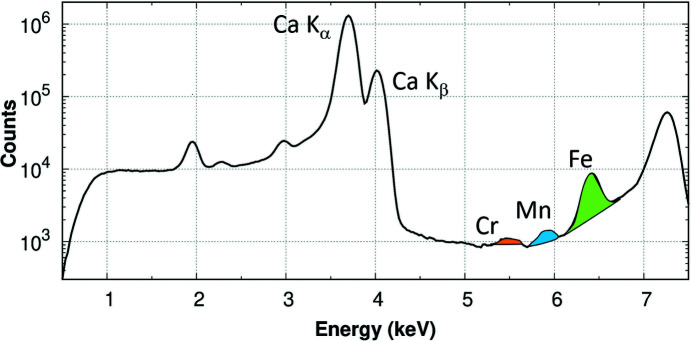
XRF spectrum of the C20 sample: the fluorescence intensities are shown on a log-scale; the Fe, Mn and Cr *K*_α_ fluorescence signals are emphasized in colour.

The samples were ground in fine powders, mixed with light-absorbing matrix (cellulose) and pressed into thin solid pellets. The pellets were then mounted in a vacuum cell, with their surface at 45° with respect to the incident X-ray beam and the detector. The sample-to-detector distance was 2–4 cm covering a solid angle of 11–3%. A raw XRF spectrum from sample C20 is shown in Fig. 4 ▸ as an example: the weakness of the fluorescence signal from elements of interest (Fe, Mn and Cr) with respect to the total fluorescence is evident. For the four samples, the fraction of the counts related to the three inspected elements (Cts_el_/Cts_tot_) was approximately: Cr, 1 × 10^−3^; Mn, 2 × 10^−3^; Fe, 8 × 10^−3^.

XAS data were acquired in energy step scanning mode using a Si (111) double-crystal monochromator. For each energy point the incident beam X-ray intensity *I*_0_ was measured by a gas-filled ionization chamber and a full X-ray fluorescence spectrum was recorder by each of the 64 detector elements as described above. The raw fluorescence signals were individually treated to identify and remove spoiled signals (saturation, spikes) while the dead-time correction has been implemented applying a not-paralyzable detection model (Ciatto *et al.*, 2004 ▸). The fluorescence signals corresponding to the *K*_α_ emission of the absorbing elements 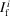 were obtained from the regions of interest (ROIs), and the absorption signal α was calculated as 

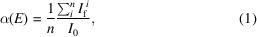

where the sum runs over the *n* active detector cells at that energy point. For each sample several spectra α^*j*^ were collected and summed up.

Additional Fe, Mn and Cr *K*-edge spectra were measured in transmission geometry on a set of reference compounds to be used for quantitative XANES analysis through linear combination analysis (LCA) (see below). The raw data were processed using the well known procedures (Bunker, 2010 ▸) for XAFS data treatment including pre-edge background subtraction, post-edge bare ion model definition, and edge jump normalization.

**Figure 5:**
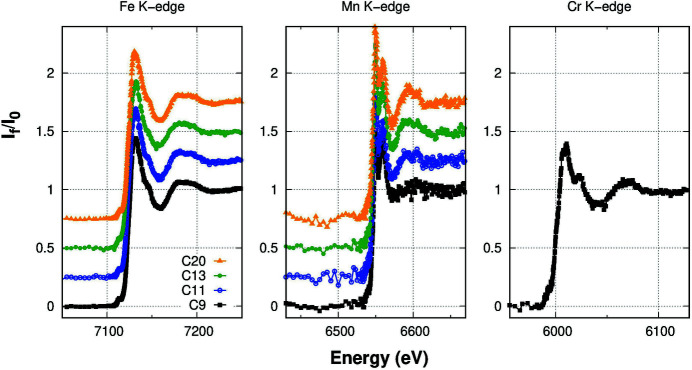
Normalized XANES spectra at the Fe, Mn and Cr *K*-edges. Spectra are vertically shifted for clarity.

The Fe *K*-edge XAFS normalized spectra of the three investigated samples are shown in the left-hand panel of Fig. 5 ▸. Fe is the most concentrated element among the ones investigated. The Fe *K*_α_ fluorescence signal corresponds roughly to 10^−3^ of the total XRF signal in each detector element; a single XAS scan in the XANES region required approximately 40 min. The normalized XANES spectra shown in Fig. 5 ▸ required roughly 2 h per sample (3–4 scans). Such an Fe concentration is quite routinely investigated at XAFS facilities and barely accessible with single-element detectors. The Fe data allowed to test the procedures for the preliminary on-the fly data treatment.

The Mn concentration in the investigated samples was approximately one order of magnitude lower than that of Fe, the Mn *K*_α_ fluorescence signal corresponding roughly to some 10^−4^ of the total XRF signal in each detector element. A larger number of energy scans (8–12 per sample) was then required to obtain suitable statistics for a quantitative XANES analysis. Each of the normalized Mn *K*-edge XAFS spectra presented in the central panel of Fig. 5 ▸ required 4–6 h of total measurement time. The statistical noise is slightly larger than for Fe *K*-edge spectra due to the lower Mn concentration and represents a suitable compromise for an appropriate quantitative XANES analysis.

The few-p.p.m. Cr concentration in the samples represents a challenge to test the limits of the multi-detector for XANES acquisition. Due to the long acquisition times and the limited beam time allocated for the experiment, the Cr *K*-edge was investigated just for one sample: the one with the lowest Cr concentration of 2.9 p.p.m. The Cr *K*_α_ fluorescence signal, being less than 10^−4^ of the total XRF signal, corresponds to a few hundred counts per second in each detector element. The spectrum presented in the right-hand panel of Fig. 5 ▸ required about 12 h, averaging 18 energy scans. Despite the higher statistical noise in the data, the main spectral features are suitable for reliable quantitative analysis to assess the Cr local coordination chemistry in the sample.

## 3. Data analysis and results

**Figure 6:**
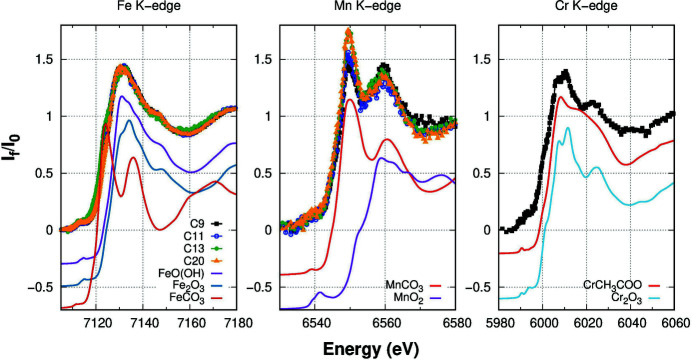
Normalized XANES spectra at the Fe, Mn and Cr *K*-edge for the samples and for significant reference compounds.

Figure 6 ▸ shows the Fe *K*-edge spectra of our samples together with those from relevant reference samples. In all our samples the Fe spectra are consistent with mainly Fe^3+^ valence state. Noticeably, the XANES spectra depict changes in the edge shoulder around 7125 eV that rises with lowering Fe concentration (*i.e.* following the order: C9, C20, C11, C13; details in Table 1 ▸). Such a shoulder seems to be related to the presence of Fe-carbonate (FeCO_3_) for which XANES depicts an evident peak at such energy. The observed trend suggests the raising of the FeCO_3_ phase fraction for the lower-concentrated Fe-samples.

The Mn *K*-edge experimental spectra depict a double-peak structure at 6550 eV and 6560 eV. Looking at the XANES spectra of reference compounds, the double peak is likely associated with an Mn-carbonate (MnCO_3_) phase. Changes among the samples mainly occur to the intensity of the main peak at 6550 eV.

The Cr *K*-edge XANES spectrum measured from sample C9 is presented in Fig. 6 ▸ compared with the Cr *K*-edge spectra from the relevant reference compounds. The double-peak shape of the white line clearly stands out around 6010 eV followed by a secondary peak around 6025 eV. These features are typical of the Cr_2_O_3_ phase, while they are absent in the other references.

To quantitatively assess the electronic state and coordination chemistry of Fe, Mn and Cr in these samples, the XANES spectra were analysed using XANES spectra from reference compounds measured in transmission at the XAFS beamline. The LCA describes the local structure of the sample as a combination of phases whose local coordination chemistry and valence state are similar to those of the references (Benfatto & Meneghini, 2015 ▸).

**Figure 7:**
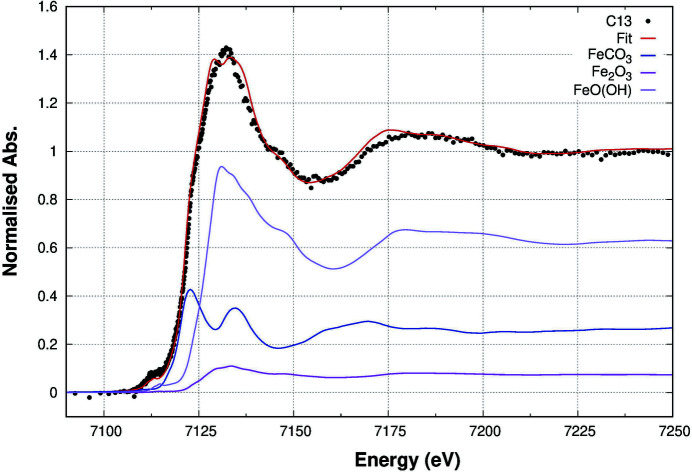
LCA results for sample C13 (Fe concentration = 130 p.p.m.) at the Fe *K*-edge. The best fit (red line) to the experimental data (black dots) is obtained by a linear combination of the different phases, reported in the plot according to their multiplicative coefficients.

After preliminary attempts, we could adequately reproduce the Fe *K*-edge XANES using three phases. The results of the analysis are presented in Table 2 ▸ for all the samples, while Fig. 7 ▸ reports the best fit (red line) to the experimental data (black dots) for sample C13, *i.e.* the one having the lowest Fe concentration. Table 2 ▸ points out that the Fe in our samples is present mostly as a carbonate phase similar to the siderite (FeCO_3_) and an Fe-oxide phase locally similar to ferrihydrite [FeO(OH)] structure. Traces of hematite are revealed in sample C13.

**Figure 8:**
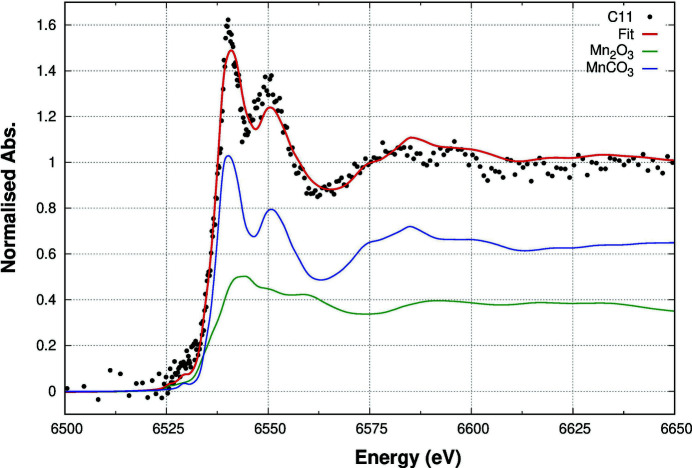
LCA results for sample C11 (Mn concentration = 13 p.p.m.) at the Mn *K*-edge. The best fit (red line) to the experimental data (black dots) is obtained by a linear combination of the different phases, reported in the plot according to their multiplicative coefficients.

The LCA of the data collected at the Mn edge revealed the presence of two phases: an Mn carbonate MnCO_3_ and an Mn^3+^ oxide similar to Mn_2_O_3_. The LCA results, reported in Table 3 ▸, show that the Mn carbonate-like phase fraction is larger for the samples with lower Mn content, while the Mn^3+^ oxide phase fraction is the lowest for the C9 sample, the one having the highest Mn concentration. The LCA best fit is presented in Fig. 8 ▸ for sample C11, having the lowest Mn concentration.

**Figure 9:**
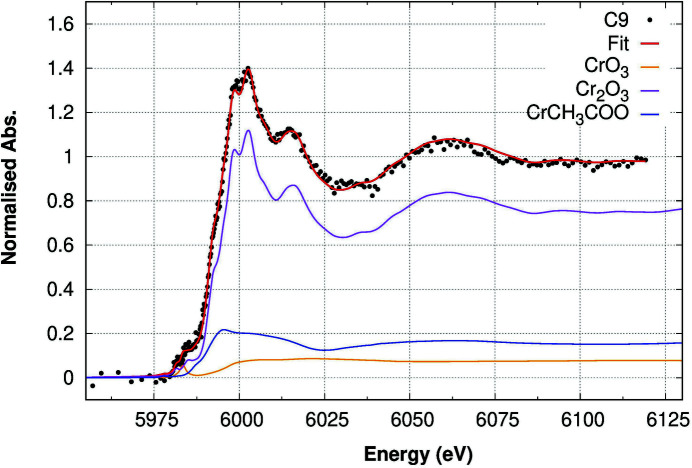
LCA results for sample C9 (Cr concentration = 2.9 p.p.m.) at the Cr *K*-edge. The best fit (red line) to the experimental data (black dots) is obtained by a linear combination of the different phases, reported in the plot according to their multiplicative coefficients.

The LCA was applied to the Cr *K*-edge XANES on the C9 sample, having less than 3 p.p.m. Cr concentration. The LCA best fit, reported in Fig. 9 ▸, required three reference spectra: a major contribution, around 77%, from the Cr^3+^ oxide phase (like Cr_2_O_3_); a Cr-carbonate phase (like CrCH_3_COO), around 16%; and a weak but statistically significant contribution from Cr^6+^ of about 7%, as shown in Table 4 ▸.

## 4. Discussion and conclusions

A general realization has emerged that there is a clear and widening gulf between the increasing photon flux delivered at the sample stage by the new-generation synchrotron radiation sources, and the capacity of the detectors in fully exploiting such abundance of photons. Off-the-shelf detectors are certainly attractive solutions for many beamlines in general and for XRF beamlines in particular. However, especially in XAFS experiments, for the thorough exploitation of their potential, a tailored detection system serving specific purposes is required. The use of multi-element detection systems opens new perspectives for X-ray research as for instance demonstrated by the MAIA detector or the Ardesia system (Ryan *et al.*, 2014 ▸; Hafizh *et al.*, 2019 ▸). Owing to their large solid angle coverage they help to reduce acquisition times thus enabling higher user throughput on the beamlines. The system described here, designed expressly for XAFS, has the ability of measuring large photon fluence rates without saturating the detector, which is of pivotal importance for diluted samples. For this purpose, a highly pixelated detector divided into 64 small elements has been designed, fabricated and tested. At the moment the detector is designed to operate with peaking times ≥0.4 µs, resulting in a maximum count rate of 2.5 × 10^5^ counts s^−1^ cell^−1^, comparable with the performance of other state-of-the-art detectors with the same peaking time. In these conditions, however, the detector is capable of handling a total count rate ≥10 × 10^6^ counts s^−1^, which to our knowledge is higher than any other detector system currently available. If required, with a modification of the acquisition scheme (analog high-pass filter instead of a CR-RC^2^ scheme) and sacrificing marginally energy resolution it is possible to reduce the minimum peaking time to the 100 ns level or below, reaching count rates of the order of 1 × 10^6^ counts s^−1^ cell^−1^, thus 64 MHz for the entire detector.

For the cooled multi-element detector described here the energy resolution, especially for low input count rates, is marginally inferior to the single-element SDD system relying on Cube technology (Cube, 2012 ▸). In the present system, it is a matter of some pick-up on the bias lines, which will be remedied in the next version of the detector. As an additional and valuable feature it is noteworthy that the spatial resolution provided by the system enables morphological studies of the sample (Billè *et al.*, 2016 ▸).

The potentialities of our detector system were presented through a case study about trace elements in rocks across stratigraphic sequences and geochemical association, a valuable tool to gain insight into geological events. Understanding the 3*d* metals mobility across oceans and their incorporation in sediments requires not only the knowledge of geochemical concentrations but also their mineralogical speciation. This work presented the use of a prototype high-performance detector to collect XANES spectra at few p.p.m. concentration for 3*d* metals Fe, Mn and Cr and providing a good quality signal, suitable for quantitative chemical speciation.

Our data show that Fe^2+^ and Mn^2+^ can form their calcite series bearing carbonate, but they also form Fe^3+^ and Mn^3+^ oxides. This suggests that Fe and Mn carbonates can get through partial oxidative dissolution with precipitation of secondary oxides. However, biological interfaces often control oxidation reactions and then two different oxidative states of trace elements can appear due to the primary carbonate formation process. Cr is mainly present as Cr^3+^ oxide with weak traces of Cr^6+^. Interestingly, the double peak in the XANES white line is consistent with Cr *K*-edge spectra of Ca_3_Cr_2_(SiO_4_)_3_ (Liu *et al.*, 2020 ▸) suggesting a major fraction of Cr in the calcium silicate environment. The well resolved XANES features suggest a relatively ordered and highly crystalline environment. Some amount of Cr^3+^ is found in a disordered atomic environment; owing to the weakness of this contribution (around 16%) it is difficult to assess the chemical speciation: Cr acetate is a candidate but the similarity of Cr *K*-edge XANES in other amorphous environments like Cr^3+^-doped glasses or oxy-hydroxides (Ohta, 2015 ▸; Liu *et al.*, 2020 ▸) prevents achieving a definitive answer. These results prove that the new multidetector system provides good quality XANES spectra from trace elements down to the p.p.m. concentration, suitable for quantitative analysis.

## Figures and Tables

**Table 1:** Concentrations (in p.p.m.) of Fe, Mn and Cr in the investigated samples (uncertainties are reported in parentheses)

	Fe (p.p.m.)	Mn (p.p.m.)	Cr (p.p.m.)
C9	400 (±20)	38 (±2)	2.9 (±0.1)
C11	130 (±1)	13 (±1)	2.9 (±0.1)
C13	130 (±1)	20 (±1)	3.5 (±0.2)
C20	210 (±10)	13 (±1)	4.5 (±0.2)

**Table 2:** Total and relative Fe concentrations of the reference compounds phases based on LCA analysis (uncertainties on the last digit are reported in parentheses)

	C9	C11	C13	C20
Ferrihydrite [FeO(OH)]	0.81 (5)	0.64 (5)	0.64 (7)	0.81 (7)
Hematite (Fe_2_O_3_)	–	–	0.07 (4)	–
Siderite (FeCO_3_)	0.19 (1)	0.36 (7)	0.26 (1)	0.19 (3)

**Table 3:** Total and relative Mn concentrations of the reference compounds phases based on LCA analysis (uncertainties on the last digit are reported in parentheses)

	C9	C11	C13	C20
Bixbyite (Mn_2_O_3_)	0.54 (7)	0.35 (5)	0.35 (6)	0.21 (5)
Rhodochrosite (MnCO_3_)	0.43 (8)	0.64 (6)	0.64 (8)	0.79 (7)

**Table 4:** Total and relative Cr concentrations of the reference compounds phases based on LCA analysis (uncertainties on the last digit are reported in parentheses)

Phase	C9
Chromium trioxide (CrO_3_)	0.07 (3)
Chromium acetate (CrCH_3_COO)	0.16 (3)
Eskolaite (Cr_2_O_3_)	0.77 (3)
